# A pilot, single-arm feasibility study of a multidimensional behavioral intervention for cognitive fatigability in multiple sclerosis: Mental Energy Boost program

**DOI:** 10.3389/fneur.2026.1823950

**Published:** 2026-05-21

**Authors:** Tamanna Islam, Jason A. Berard, Abby Ryan, Marcia Finlayson, Lara A. Pilutti, Sarah Morrow, Lisa A. S. Walker

**Affiliations:** 1 Department of Psychology, University of Ottawa, Ottawa, ON, Canada; 2 Department of Neuroscience, Ottawa Hospital Research Institute, Ottawa, ON, Canada; 3 University of Ottawa Brain and Mind Research Institute, University of Ottawa, Ottawa, ON, Canada; 4 Ottawa Valley Family Health Team, Almonte, ON, Canada; 5 School of Rehabilitation Therapy, Queen's University, Kingston, ON, Canada; 6 Interdisciplinary School of Health Sciences, University of Ottawa, Ottawa, ON, Canada; 7 Calgary Multiple Sclerosis Program, Cumming School of Medicine, Department of Clinical Neurosciences, Calgary, AB, Canada

**Keywords:** behavioral intervention, cognitive fatigability, feasibility study, multiple sclerosis, traffic light decision framework

## Abstract

**Background:**

Cognitive fatigability (CF), the decline in performance following sustained cognitive effort, remains less understood in people with multiple sclerosis (PwMS) than subjective fatigue, and no behavioral interventions have yet specifically targeted CF in PwMS.

**Purpose:**

This study assessed the feasibility of an 8-week, group-based, video conference-delivered behavioral intervention for CF in PwMS to determine if proceeding to a full-scale randomized controlled trial (RCT) is advised.

**Methods:**

The program was adapted from a validated teleconference-based program for fatigue in MS, incorporating additional components targeting factors known to contribute to CF, and informed by investigators' clinical expertise and perspectives of PwMS from a needs assessment survey. Following the sessions, focus group discussions took place to gather participant's feedback. Feasibility was assessed by tracking eligibility, recruitment, adherence, completion, acceptability, and treatment fidelity, using a traffic-light framework (GREEN = proceed to RCT; AMBER = amend before proceeding to RCT; RED = do not proceed to RCT) to determine whether modifications are needed before a full trial, with thresholds based on prior feasibility studies.

**Results:**

Of 39 PwMS screened from an MS clinic sample, 21 (54%) were eligible and 18 were enrolled (86%), reflecting a high recruitment uptake. Recruitment was completed over 9 months. Satisfaction with the overall program was high, with 92% respondents rating their satisfaction >5 on a 10-point scale (1 = completely dissatisfied; 10 = completely satisfied). Perceived helpfulness of sessions was also high (92% scoring ≥ 24/40). Per session attendance ranged from 59% to 94% (Mean = 81.62%), with an 89% (*n* = 16) completion rate, and a 94% (*n* = 15) focus group attendance rate. Treatment fidelity (i.e., coverage of manualized content) was high, with the facilitator adhering to the session length in 79% of sessions.

**Conclusion:**

Most feasibility indicators met “GREEN” criteria, supporting progression to a full trial. Recruitment period duration was the only indicator that fell within the AMBER zone, indicating a need for procedural adjustments before a full trial. No indicator fell in the RED zone.

## 1 Introduction

Fatigue is among the most prevalent and debilitating symptoms of MS, reported by up to 90% of people with MS (PwMS) (1), regardless of disease phenotype. MS-related fatigue is commonly described as an overwhelming feeling of tiredness, reduced energy, diminished motivation, and difficulty in concentrating and can only be assessed by self-report (2, 3). In addition to fatigue, fatigability is also frequently reported in MS (4). Although conceptually different, emerging evidence suggests a reciprocal influence between fatigue and fatigability in MS (5). Unlike fatigue, which reflects a subjective experience, fatigability can be operationalized as either subjective (perceived) or objective (performance based) (2). Subjective fatigability refers to self-reported performance decline in relation to past, present, or anticipated work capacity in the context of standardized activities, whereas objective fatigability is quantified through measurable declines in performance over time (2).

Cognitive fatigability (CF), more specifically, has only recently gained increased attention. CF refers to a decline in cognitive performance following sustained cognitive effort and remains less understood in PwMS than subjective fatigue (6, 7). Consistent with the broader fatigability framework, CF in MS can also be conceptualized either subjectively using self-report (e.g., the Pittsburgh Fatigability Scale) (8, 9) or objectively through performance-based measures that assess changes in cognitive performances over time [e.g., the Paced Auditory Serial Addition Test (PASAT)] (7, 10). The outcome of the present study is objectively measured CF using the PASAT.

Although both fatigue and CF adversely affect daily functioning (e.g., employment, social functioning, etc.) and quality of life in PwMS (11–13), CF is particularly clinically relevant due to its impact on the ability to sustain cognitive performance in everyday contexts. Despite this, the existing intervention literature has largely focused on treatments targeting subjectively evaluated fatigue. These interventions fall into three approaches: pharmacological, procedural (e.g., light therapy, biofeedback, neuromodulation), and behavioral approaches (14–16). Our group's systematic review identified a clear paucity of interventions specifically designed to target objectively measured CF in MS (17). While recent studies have begun to explore procedural (e.g., transcranial direct current stimulation) (18) and pharmacological (e.g., Fampridine) (19) approaches to improving CF, behavioral interventions explicitly designed to address CF are currently lacking. Given that CF is associated with modifiable factors such as mood, sleep quality, physical activity, and contextual factors (20–23), and behavioral interventions have previously been effective for MS-related fatigue (16), behavioral approaches targeting these CF contributors provide a promising avenue for intervention. This gap provided the rationale for the current project.

Existing behavioral interventions for MS-related fatigue primarily address subjective fatigue and do not explicitly focus on the multidimensional contributors to CF. Consequently, a recent needs assessment survey conducted by our research group indicated that those experiencing CF continue to report unmet needs across most contributory factors of CF (21), including sleep quality, fatigue, cognitive impairment, depression and contextual factors (22). To address these ongoing needs, the present study aimed to evaluate the feasibility of a multidimensional behavioral intervention designed to improve CF in PwMS, providing a foundation for a future definitive randomized control trial (RCT).

As a pilot feasibility trial, this single arm study evaluated core feasibility indicators, including eligibility rate, recruitment uptake and duration, adherence, completion, acceptability, and treatment fidelity. Attendance at a post-intervention focus group discussion was also assessed for additional information. Feasibility outcomes were assessed using a traffic-light decision framework, to inform progression to a future full-scale RCT, previously applied in feasibility studies (24, 25). In this framework, **GREEN** indicates progression to a full trial without any changes, **AMBER** indicates that progression is possible but requires correctable adjustments, and **RED** indicates that progression should not occur due to issues that cannot be readily addressed (25). The progression method, GREEN (go), AMBER (amend), RED (stop), was originally introduced and conceptualized as a traffic light system by Avery et al. (26). Ideally, all feasibility indicators should fall within the **GREEN** zone; however, **AMBER** outcomes are also acceptable and equally informative, as they highlight specific areas where modifications may be needed to support progression to a full RCT. Consistent with this framework, the aim of the present study was to determine whether progression to a full RCT should occur without modifications (GREEN), with modifications (AMBER), or not at all (RED).

## 2 Methods

To ensure replicability, the intervention is described in accordance with the Intervention Description and Replication (TIDieR) checklist (27), incorporating elements specific to rehabilitation trials from the TIDieR-Rehab checklist (Supplementary Appendix A) (27–30). The study employed a pre-post design and was approved by the Ottawa Health Science Network (20240351-01H) and the University of Ottawa Health Sciences and Science (H-07-24-10612) Research Ethics Boards.

### 2.1 Intervention description

Our group developed a multi-dimensional behavioral intervention to target CF in PwMS, the *Mental Energy Boost* program, using established behavioral and educational principles, drawing on theory and evidence to guide its structure, content, and delivery as outlined in a previously published protocol paper (6). The program was grounded in four theoretical models. First, Social Cognitive Theory (SCT) recognizes that learning occurs within a social context involving the interaction between the person, their environment and behavior (31). The goal of SCT in this program was to assist people in regulating their behavior and reinforcing positive changes to help them achieve their personal goals related to CF management. Second, Psychoeducation Group Development Theory (32), informs interventions that are delivered by trained professionals, and that integrate psychotherapeutic principles and education about specific topics. Thus, the program was delivered by a trained facilitator with prior clinical experience that enhanced their ability to interpret the material in a meaningful way for participants. Third, principles of Cognitive Behavioral Therapy (CBT) were integrated to demonstrate to participants that their emotions, thoughts and behaviors are inter-linked (33). By addressing some of the myths people have about cognitive fatigability participants can learn new ways of thinking and behaving that can lead to improved outcomes. Finally, a modified version of Bloom's Taxonomy ensured that the program content was sequenced over 8 sessions to begin with foundational remembering of information (e.g., learned facts) and eventually progressed to higher order cognitive skills (e.g., synthesizing information and finding ways to integrate and apply it in their own life context) (34, 35). Each session was designed to build upon the previous session, fostering a comprehensive understanding and application of strategies to manage CF. Such an approach fosters confidence that participants can independently apply the learned principles and sustain this post-intervention.

The *Mental Energy Boost* program was also adapted from a previously validated teleconference-delivered program for MS-related fatigue (16), taking elements of this established program for MS and further integrating additional components to address factors known to contribute to objectively measured CF (21). While objective CF and subjective fatigue are distinct concepts (20), they share some common contributing factors (e.g., contextual factors) (20, 21). The program was also informed by clinician expertise, and refined using findings from a needs assessment survey of PwMS (22). The resulting intervention is an 8-week, group-based, videoconference-delivered program targeting key domains relevant to CF, through structured sessions, homework activities, and goal setting.

Goal setting was an important component that allowed participants to monitor individualized progress as the sessions progressed (36, 37). Standardized facilitator and participant manuals were developed. Both manuals included general information about how to utilize the videoconference platform, contact information for technical and study assistance, as well as all content for each 70-min session, ensuring standardization of the intervention and fidelity in delivery across the three groups. The facilitator manual additionally included information on the theoretical underpinnings of the program, a preparation checklist, and technical information about the videoconference platform.

**Figure 1:**
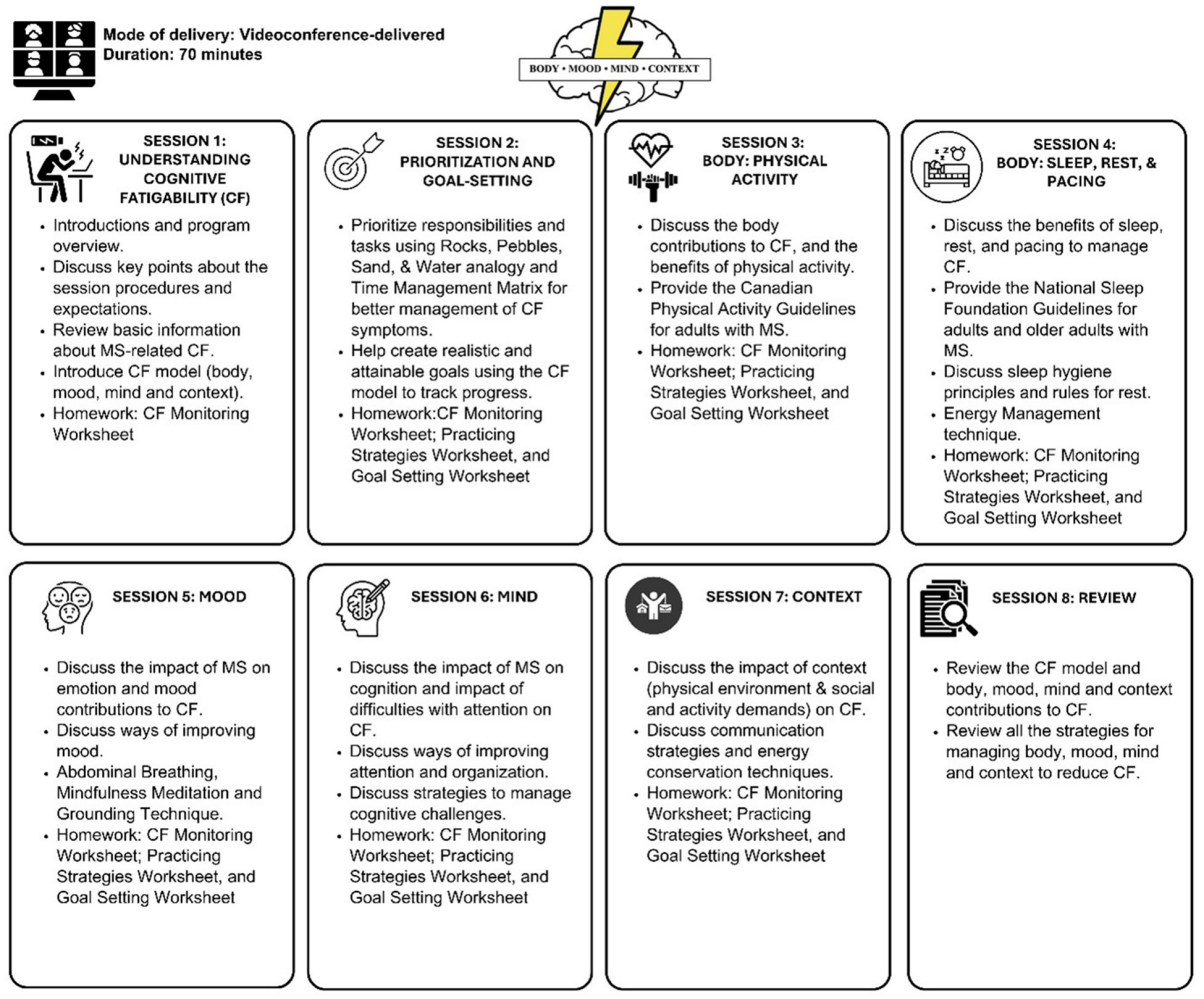
Overview of sessions.

To address the multidimensional nature of CF, and to ensure the material was accessible to participants, the *Mental Energy Boost* program content was organized into four easy-to-understand thematic domains: body (sleep, physical activity), mood (depression and anxiety), mind (cognitive contributions), and context (pacing, communication). Figure 1 provides an overview of the eight sessions of the program, including session content, key activities, and duration. Participants completed homework assignments between sessions that helped them monitor their CF and associated contributing factors, while also allowing them to establish and refine personalized goals to improve motivation and ensure the information was personally relevant.

### 2.2 Participants

To accommodate an anticipated attrition rate of 25% (38, 39), we recruited 21 PwMS who met the inclusion criteria from the Ottawa Hospital MS Clinic. The primary inclusion criterion was the presence of objective CF on the PASAT, defined using established normative data as ≥1.5 standard deviations below the mean (7). Additional inclusion criteria were: (a) English-speaking; (b) between 18 and 65 years of age; (c) EDSS <6.0, (d) relapse and steroid free in the past 30 days; (e) purposeful exercise ≤ 2 days per week for 30 min; (f) asymptomatic (i.e., no signs or symptoms of acute or uncontrolled cardiovascular, metabolic, or renal disease) based on the Get Active Questionnaire; (g) sufficient visual function to complete cognitive tasks (e.g., no scotomas) and (h) access to an internet-enabled device to participate in the intervention (6). Exclusion criteria included: (a) other neurological, medical or psychiatric conditions that might impede cognition (e.g., traumatic brain injury, learning disability, ADHD), excluding depression and anxiety; (b) current dementia; (c) substance use or dependence disorder; and (d) hearing impairment that would interfere with the ability to effectively take part in the videoconference sessions (6).

### 2.3 Outcome measures

Primary outcomes focused on intervention feasibility. Feasibility was assessed using a predefined set of indicators within a “traffic light” framework, including eligibility, recruitment uptake, duration of the recruitment period, intervention acceptability, adherence, completion rate, treatment fidelity, and attendance at the post-intervention focus group discussion (see the predefined criteria described in Table 1). These outcomes will inform decisions regarding progression to a future RCT. These data were interpreted using the traffic-light decision framework. Further, qualitative data were obtained from the open-ended questions on the evaluation form completed during the follow-up assessment session, providing additional insights into participants' experiences and perceptions of the intervention.

**Table 1:** Feasibility criteria developed for the Mental Energy Boost study using traffic light thresholds.

Feasibility outcomes	Explanation	GREEN zone (proceed)	AMBER zone (amend)	RED Zone (do not proceed)	Results	Resulting zone
Eligibility (42)	The number of screened participants who can take part in the study, whether they later agree to or not	≥25%	>10% and <25%	≤ 10%	53.84%	GREEN
Recruitment uptake (24)	Percent of eligible participants recruited	≥35%	>20% and <35%	≤ 20%	85.71%	GREEN
Duration of the recruitment period (43, 44)	The timeframe during which participants are enrolled or recruited into the program	≤ 5 months	>5 months and <2 years	≥2 years	~9 months	AMBER
Intervention acceptability (25, 42)	Evaluate respondents' (i) satisfaction and (ii) perceived effectiveness of the strategies employed in the program	(i) ≥75% of respondents score >5 on overall satisfaction.	(i) ≥30% and <75% of respondents score >5 on overall satisfaction.	(i) <30% score >5 on overall satisfaction.	(i) 92.31% of respondents scored >5 on overall satisfaction.	GREEN
		(ii) ≥50% of respondents score a total of ≥24 across all 8 evaluation items	(ii) ≥30% and <50% of participants score a total of ≥24 across all 8 evaluation items	(ii) <30% of respondents score a total of ≥24 across all 8 evaluation items	(ii) 92.31% of respondents scored a total of ≥24 across all 8 evaluation items	GREEN
Adherence	Attendance at sessions	≥50% of participants attend ≥80% of sessions	≥30 and <50 of participants attend ≥80% of sessions	<30% of participants attend ≥80% of sessions	58.82%, ranging from 59% to 94%	GREEN
Completion rate (46)	The number of participants who complete the follow-up assessment	≥70%	>50% and <70%	≤ 50%	88.89%	GREEN
Treatment fidelity (45, 46)	Evaluate therapists' views on the intervention delivery in terms of: (i) The extent to which all content from the participant manual was covered during sessions (ii)Whether the material for each session was delivered within the designated 70-min timeframe	(i) All participant manual content was covered in ≥70% of the sessions	(i) All participant manual content was covered in > 40% and <70% of the sessions	(i) All participant manual content was covered in ≤ 40% of the sessions	(i) All content covered in 100% of the sessions	GREEN
		(ii) All participant manual content was delivered within the timeframe in ≥70% of the sessions	(ii) All participant manual content was delivered within the timeframe in >40% and <70% of the sessions	(ii) All participant manual content was delivered within the timeframe in ≤ 40% of the sessions	(ii) All content delivered within the timeframe in 79.17% of the sessions	GREEN
Post-intervention focus group discussion (46)	Attendance at focus group discussion	≥ 70% of participants attend post-intervention focus group discussion	>50% and <70% of participants attend post-intervention focus group discussion	<50% of participants attend post-intervention focus group discussion	93.75% of participants attended	GREEN

### 2.4 Procedures

An exploratory, single-arm, within-subjects repeated-measures design was implemented. Participants who consented to be contacted for research purposes were identified via electronic health records of the local MS Clinic. Their preferred language and age were confirmed, and MS diagnosis and EDSS score were verified. All individuals who met preliminary criteria (English-speaking; between 18 and 65 years of age; EDSS <6.0) were emailed an invitation to participate and were instructed to contact the research team if interested. Those who expressed interest completed an in-person screening session with the research assistant (TI). During this visit, participants completed an initial screening questionnaire, including the Get Active Questionnaire (GAQ), followed by the PASAT to determine if they exhibited statistically significant objective CF (7). Screening took place surrounding their next scheduled clinic appointment or at a separate visit, depending on participant preference. Eligible participants subsequently provided written informed consent at the same in-person screening visit.

Based on prior research recommending that feasibility in MS studies can be determined with 12 to 15 participants (40, 41), we selected a target sample size to ensure at least 15 individuals in the final sample. The intervention was then delivered via videoconference in three groups of approximately 5–7 participants each (6). Three groups were chosen to facilitate interaction within a manageable group format, ensuring participants had adequate time for questions and meaningful engagement during each 70-min session, while also allowing scheduling flexibility.

For enrolled participants, the intervention was delivered once weekly for 8 consecutive weeks by a licensed Occupational Therapist (AR), who was trained and supervised by a Clinical Neuropsychologist (LW). An exception occurred for session 7 of group 3, which was delivered by the Principal Investigator (LW), given that AR was unavailable due to illness. Prior to implementing the intervention, the facilitator manual was reviewed by AR, and additional input was sought. The videoconference platform used was Microsoft Teams (MS Teams). This platform was chosen to ensure compliance with privacy standards. Each session lasted approximately 70 min, consistent with the previously validated teleconference program for MS-related fatigue from which our intervention was adapted (16). This included a 10-min mid-session break to help mitigate CF induced by session content. Participants received the participant manual and homework assignments via email. Additionally, a printed version of the homework assignments was also provided during the baseline assessment to accommodate preferences for paper-based completion.

Intervention sessions were held on weekdays, with participants given the option to attend one of two different late afternoons based on the facilitator's availability. Final session times were then determined according to the majority preference within each group. While this schedule accommodated most participants, it may have been less convenient for those working full-time, potentially contributing to attendance variability.

During all intervention sessions, a research assistant (TI) was present to address any technical issues and to display the participant manual and homework materials using the MS Teams screen-sharing function, enabling participants to visually follow along with session content as it was being discussed. Between sessions, participants completed structured, but individualized, homework assignments designed to help them apply the strategies introduced each week, with the goal of enhancing self-awareness of factors contributing to their CF and integrating management techniques into daily life. In line with the underlying theoretical models guiding the intervention, participants were also encouraged to actively work toward their individualized goals identified in the preceding session, and to refine these goals over time as they implemented strategies between sessions. Participants were contacted via email the day before each session to ask whether they had completed the homework assignment for that week. No justification or additional explanation was required for their response. In the event that participants did not reply to the email, the research assistant followed up at the end of the session to record whether they had completed the homework, and their responses were recorded accordingly.

Participants who missed a session were offered an abbreviated make-up session with the research assistant to ensure continuity and coverage of all intervention content. All sessions were recorded with participants' consent and in accordance with the study's ethics approval and recordings were reviewed by the principal investigator (LW). Prior to each session, the research team (LW, AR, and TI) met for a team huddle to review the upcoming session content and provide strategies to address any issues identified in the previous session.

To ensure fidelity to the intervention protocol and provide feedback on each session, the group facilitator completed a Facilitator Feedback Checklist to document their impressions. This checklist identified: what went well, what did not go well, suggestions for improvement, and any other comments or considerations for the next session (e.g., did anything unexpected happen?). The principal investigator (LW), or another covering Clinical Psychologist, was available to provide on-call clinical support throughout the study and was available on an as-needed basis for all screening, baseline, and follow-up sessions to address any clinical emergencies. Treatment fidelity was assessed to ensure consistent delivery of the intervention by the therapist across all sessions and groups. Fidelity evaluation was focused on two key dimensions: (i) coverage of all content from the participant manual based on the Facilitator Feedback Checklist and (ii) adherence to the designated 70-min session duration, verified using recorded start and end times of each session (Table 1).

### 2.5 Data collection

#### 2.5.1 . Intervention metrics

Data on participant adherence, including make-up sessions, engagement through participation, and completion of homework, were recorded on a weekly attendance sheet by the research assistant.

The evaluation form administered during the follow-up assessment included a single item assessing overall satisfaction using a scale of 1 (completely dissatisfied) to 10 (completely satisfied). For feasibility purposes, overall satisfaction was defined *a priori* as the proportion of participants rating their satisfaction >5 (which indicates participants were more satisfied than dissatisfied), with predefined thresholds. The evaluation form also included items assessing perceived helpfulness of each of the 8 sessions. Each item was rated on a 5-point Likert scale ranging from 1 (not helpful) to 5 (extremely helpful). In addition, the evaluation form contained several open-ended questions, allowing participants to describe their experiences with the program in their own words, rather than selecting from predefined options.

#### 2.5.2 . Focus group discussion

All participants in each group who completed the intervention were invited to participate in a focus group during the week after their last intervention session. The discussion took place using Microsoft Teams and lasted between 90 and 120 min. During these meetings, the moderators (LW and TI) explained the discussion format and facilitated the discussion among the participants using a semi-structured discussion guide composed of open-ended questions to identify participant perspectives. Participants were asked to be honest and forthcoming, and both positive and negative feedback was encouraged. They were also encouraged to feel free to agree or disagree with other participants and reassured that all perspectives were valued. With participants' consent, each focus group discussion session was recorded and transcribed verbatim. Recordings and transcripts will be reviewed, and results of a thematic analysis will be reported in a separate paper. For the current feasibility analysis, only participant attendance at the focus group discussions was examined.

### 2.6 Analyses

Sociodemographics were collected using a demographic questionnaire and analyzed using frequencies, means, and standard deviations (SDs). Preliminary feasibility was assessed using descriptive and qualitative statistics. Retention, adherence and completion rates, participants' satisfaction ratings (acceptability), and treatment fidelity were analyzed using descriptive statistics according to the traffic light framework described above. Feasibility thresholds were established *a priori* based on results from earlier pilot feasibility studies in MS and other chronic health conditions (24, 42–46) (Table 1). For instance, the completion rate threshold (i.e., ≥70% of participants completing baseline and follow-up assessments) was informed by progression criteria reported by Artom et al. (46) in a behavioral intervention for fatigue in people with inflammatory bowel disease (IBD).

## 3 Results

### 3.1 Participant characteristics

Of the 21 participants who met the eligibility criteria, 18 participants (15 female and 3 male) were enrolled, as 2 participants were lost to follow-up, and 1 withdrew prior to enrollment. Participants ranged in age from 26 to 65 years (mean age = 43.83, SD = 10.92). Most were diagnosed with relapsing MS (RMS) (89%), with an average disease duration of 9.06 years (SD = 7.66). The majority were married and living with families, were employed, had completed college or other non-university diploma programs, and were residents of suburban communities (Table 2).

**Table 2:** Characteristics of the participants.

Sociodemographic characteristics	
Variable	Mean (SD)>^*^or *N* (Valid %)^∧^
Age	43.83 (10.92)^*^
Sex assigned at birth	
Female	15 (83.3%)^∧^
Male	3 (16.7%)^∧^
Gender Identity	
Woman	15 (83.3%)^∧^
Man	3 (16.7%)^∧^
Ethnicity	
Indigenous Canadian	1 (5.6%)^∧^
Caucasian-North American origins	5 (27.8%)^∧^
Caucasian-European origins	11 (61.1%)^∧^
Other (Latin/Europe)	1 (5.6%)^∧^
Marital Status	
Single/never married	3 (16.7%)^∧^
Married/common law partner	14 (77.8%)^∧^
Divorced/separated	1 (5.6%)^∧^
Current Employment Status	
Full-time/part-time paid	12 (66.7%)^∧^
Retired	2 (11.1%)^∧^
Unable to work due to illness or disability	4 (22.2%)^∧^
Highest level of education attained	
University-PhD or equivalent	1 (5.6%)^∧^
University-Master's degree	2 (11.1%)^∧^
University-Bachelor's degree	4 (22.2%)^∧^
College or other non-university diploma	8 (44.4%)^∧^
Completed secondary school/high school diploma	2 (11.1%)^∧^
Partial secondary school/high school or less	1 (5.6%)^∧^
Use of assistive equipment for mobility	
None	15 (83.33%)^∧^
Cane/crutch	4 (22.22%)^∧^
Walker	3 (16.67%)^∧^
Current living situation	
Alone	2 (11.1%)^∧^
With roommate/partner	5 (27.8%)^∧^
With family members	11 (61.1%)^∧^
**Clinical Characteristics**	
**Variable**	**Mean (SD) or** ***N*** **(Valid %)**
Disease duration (years)	9.06 (7.66)^*^
Types of MS	
RMS	16 (88.9%)^∧^
PPMS	1 (5.6%)^∧^
MS type not yet identified	1 (5.6%)^∧^
EDSS indicated as Median, (Range)	2 (0–3.5)

RMS, relapsing MS; PPMS, primary progressive MS; SPMS, secondary progressive MS; EDDS, expanded disability status scale. * is Mean (SD) and the ∧ symbol is Valid %.

### 3.2 Eligibility

**Figure 2:**
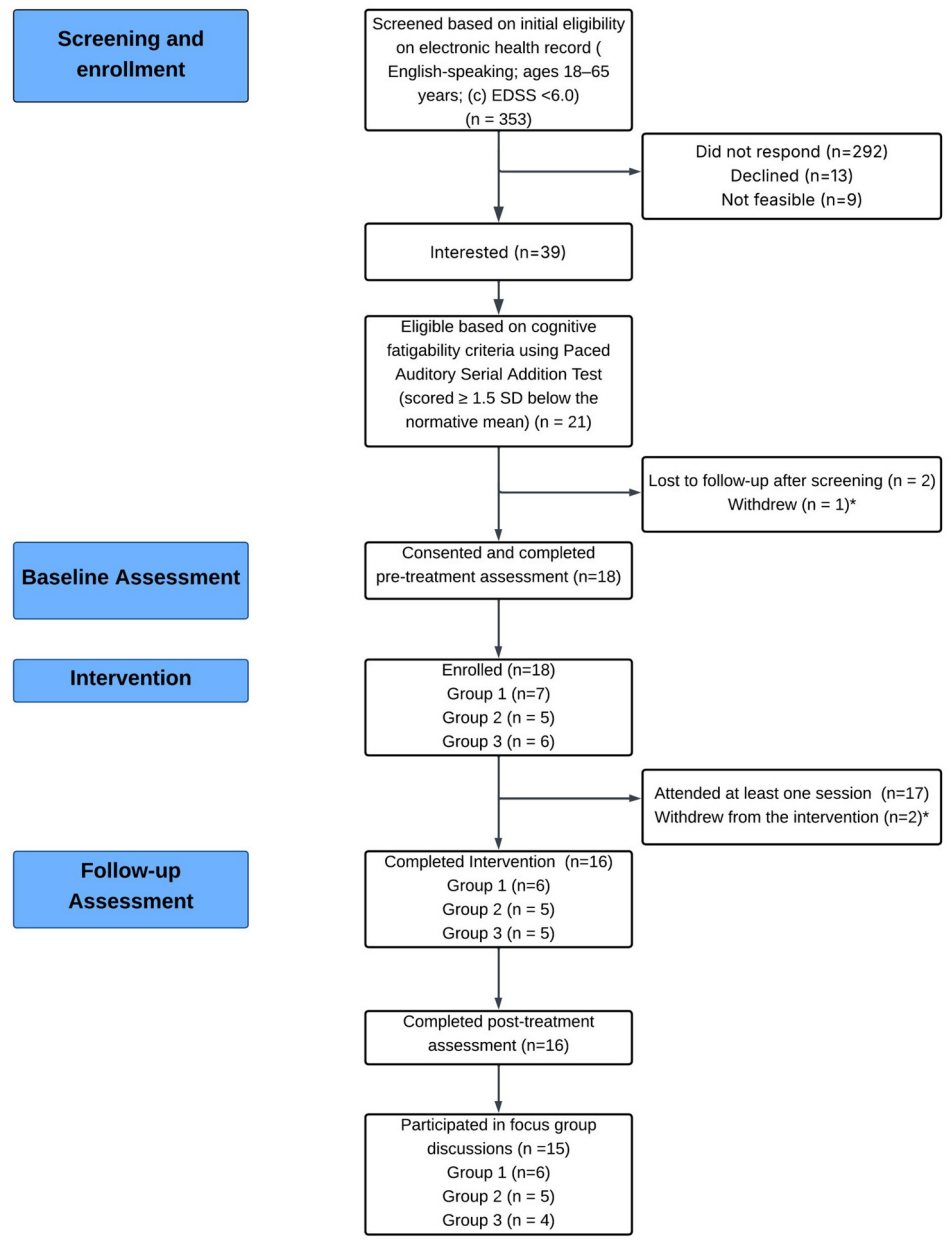
Consort diagram of participant progression.

A CONSORT flow diagram for the study is provided in Figure 2, illustrating progression through the study stages (47).

Thirty-nine PwMS completed the screening, of whom 21 met eligibility criteria, yielding an eligibility rate of 53.84%. This rate fell within the GREEN zone.

### 3.3 Recruitment uptake

Among 21 eligible participants, 18 consented and completed the baseline assessment, yielding a recruitment uptake of 85.71%. This fell within the GREEN zone.

### 3.4 Duration of the recruitment period

Recruitment for the three groups was completed over a period of 9 months, which fell within the AMBER zone.

### 3.5 Intervention acceptability

**Figure 3:**
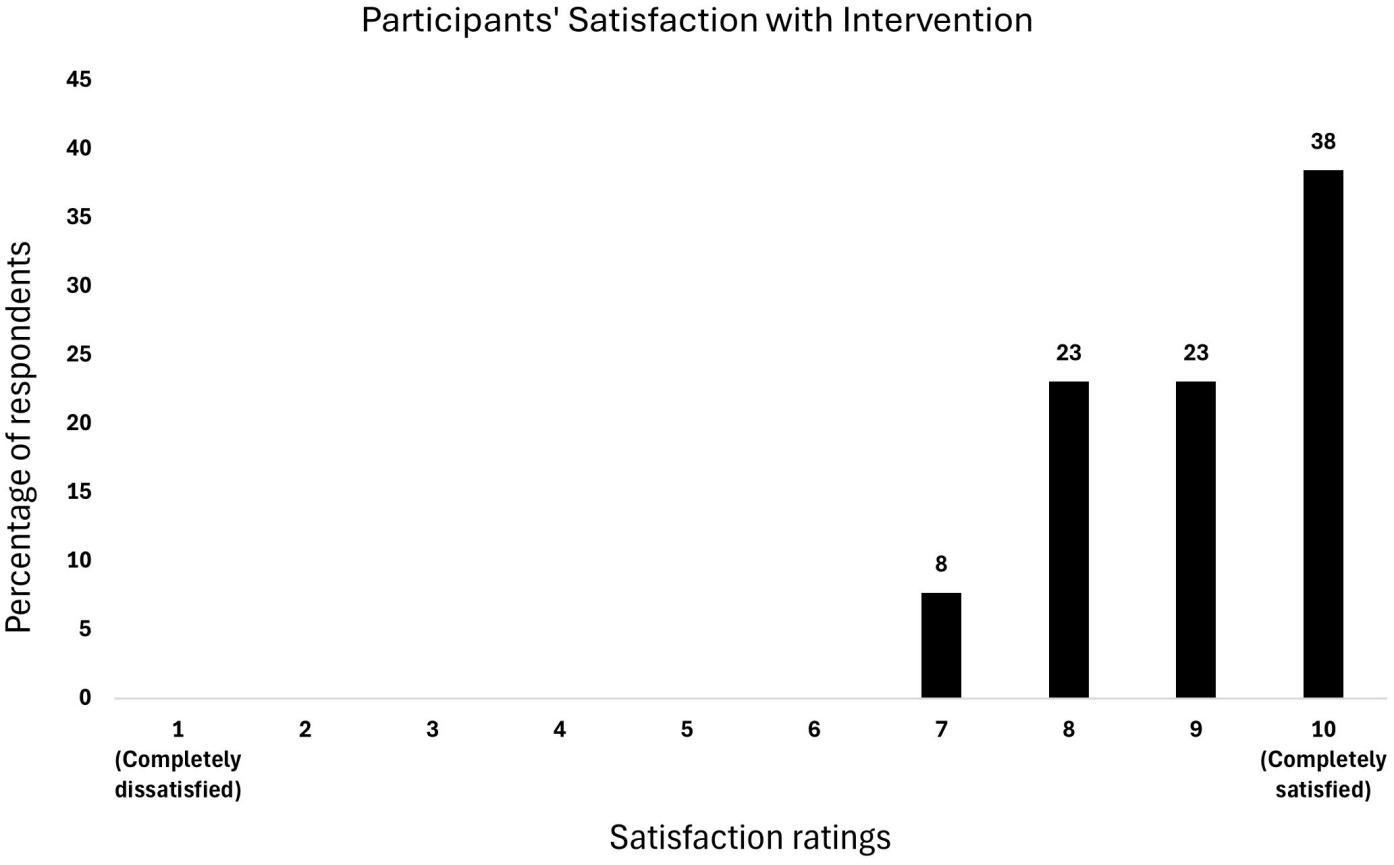
Percentage of respondents reporting satisfaction with the Mental Energy Boost program.

Of the 13 respondents who completed the evaluation form, 12 rated their satisfaction ≥5, while 1 respondent did not provide any rating, resulting in a satisfaction rate of 92.31% respondents scoring >5 (Figure 3). This corresponded to the GREEN zone.

**Figure 4:**
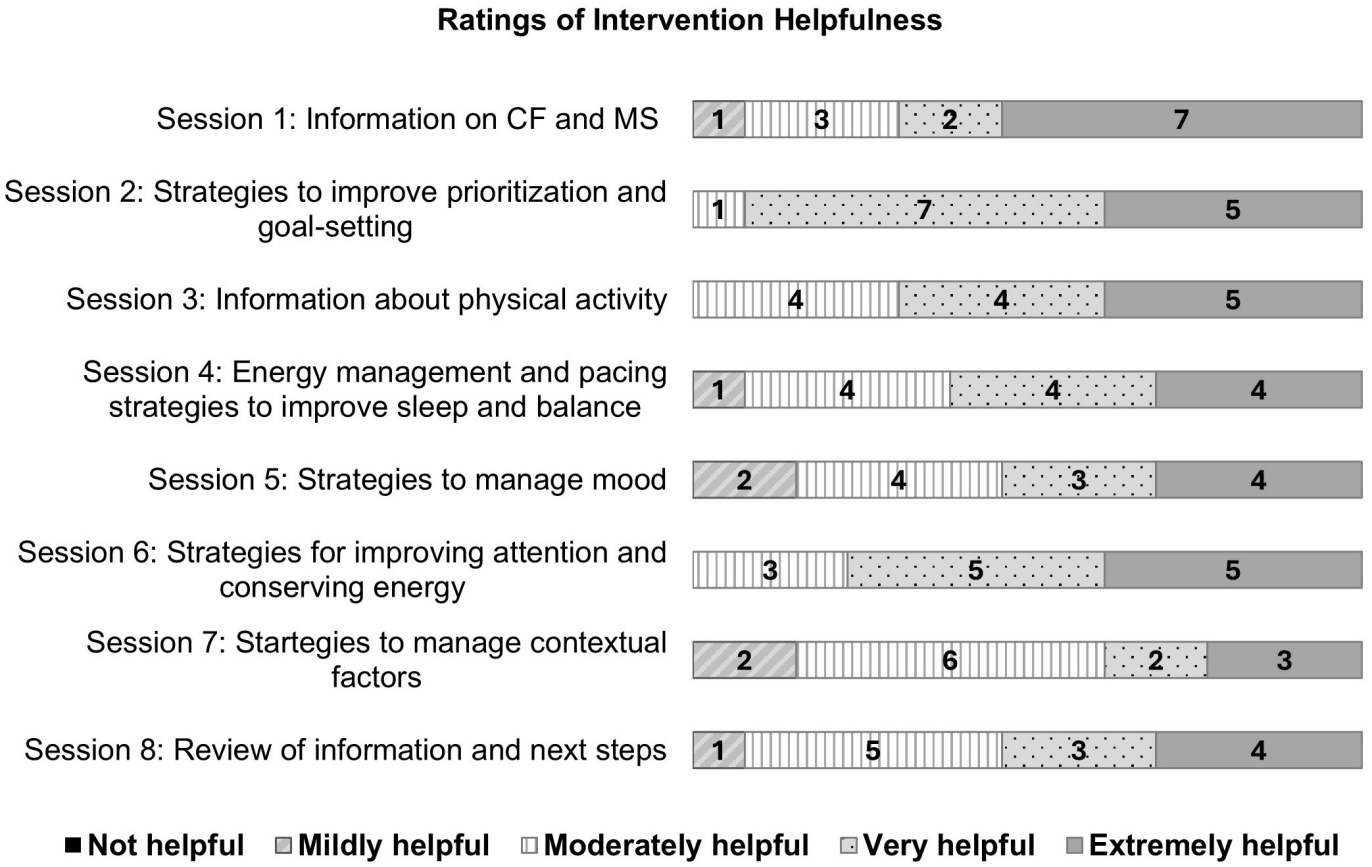
Participants' perceived helpfulness of the Mental Energy Boost intervention content.

As illustrated in Figure 4, participants rated the perceived helpfulness of the various components of the intervention. Based on thirteen responses, 12 participants scored a total of ≥24 across all 8 items, indicating that 92.31% found the content helpful across sessions. This fell within the GREEN zone.

The open-ended responses from the evaluation form are summarized in Table 3.

**Table 3:** Participant feedback on open-ended evaluation questions about the program.

Item	Summary of responses
MOST useful topic/strategy	•Energy conservation strategies (*n =* 6) •Physical activity (*n =* 4) •Mindfulness (*n =* 3), •Mood-management strategies (*n =* 3) •Goal setting (*n =* 2) •Understanding cognitive fatigability (*n =* 1) •Use of homework worksheets (*n =* 1).
Strategies adopted since the program	•Resting (*n =* 5) •Energy budgeting (*n =* 4) •Pacing (*n =* 3) •Grounding and breathing exercise (*n =* 2) •Movement/exercise/mild-to-moderate physical activity (*n =* 2) •Prioritization (*n =* 2) •Sleep hygiene (*n =* 1) •Self compassion (*n =* 1) •Consistent daily routine (*n =* 1) •Daily use of homework worksheets (n = 1)
Impact of the program on managing cognitive fatigability (CF) daily	•Increased awareness of cognitive fatigability and its triggers/factors (*n =* 9) •Improved day-to-day cognitive fatigability management using learned strategies (*n =* 9) •Decreased cognitive fatigability (*n =* 1). •Reduced feelings of isolation (*n =* 1) •Improved ability to communicate signs of cognitive fatigability with others (*n =* 1) •Reinforcement of previously known strategies (*n =* 1).
LEAST useful topic/strategy	•Context-related strategies (*n =* 4), •Attention-related strategies (e.g., LEAP—**L**isten actively, **E**liminate distractions, **A**sk questions, **P**araphrase) (*n =* 2) •Sleep strategies (*n =* 2) •Mood-management strategies (*n =* 2), and •Prioritization tools (e.g., Rocks, Pebbles, Sand, and Water analogy as well as Time Management Matrix) (*n =* 1). •Weekly addition of new strategies perceived as overwhelming (*n =* 1).
Suggestions for additional topics	•Nutrition (*n =* 2) •Strength training (*n =* 1) •Assertiveness (*n =* 1) •Strategies for discussing cognitive fatigability with employers, (e.g., employee rights, language for requesting support, and concrete examples of accommodations) (*n =* 1). •Educational resources for family/friends (*n =* 1). •Discussing the benefits of relaxation/meditation (*n =* 1).
Feedback on administrative and technical aspects of the program	•No concerns (*n =* 7) •Responsive, and clear (*n =* 3) •Appropriate session length and manageable homework (*n =* 1) •Session timing challenges (e.g., dinner/evenings) (*n =* 2). •Effective technology (*n =* 1). •Well-facilitated session (*n =* 1)
Suggestions for improving the program	•In-person options for better focus and interaction (*n =* 2) •Follow-up sessions for sharing experience (*n =* 1) •Move sessions 6 (Mind) and 7 (context) earlier (*n =* 1) •Extend program with implementation/coaching weeks (*n =* 1) •Hard copy for intervention manual (*n =* 1). •Greater emphasis on homework and sharing (*n =* 1) •Allow more time between strategies (*n =* 1) •Increase participant interaction and sharing of MS experience (*n =* 1) •Reduce reliance on reading directly from the manual, and more active presentation of content (*n =* 1).
Source of information about the program	•Through study team communications (e.g., emails) (*n =* 8) •Information provided in the clinic waiting room (e.g., flier or clerical staff) (*n =* 7).
Additional comments	•Valued and validating experience (e.g., learning from experts and peers) (*n =* 1) •Follow-up survey to support review and reassessment of strategies (*n =* 1) •Gradual tapering of sessions (*n =* 1) •Continued support to sustain strategy use (*n =* 1).

### 3.6 Adherence

**Figure 5:**
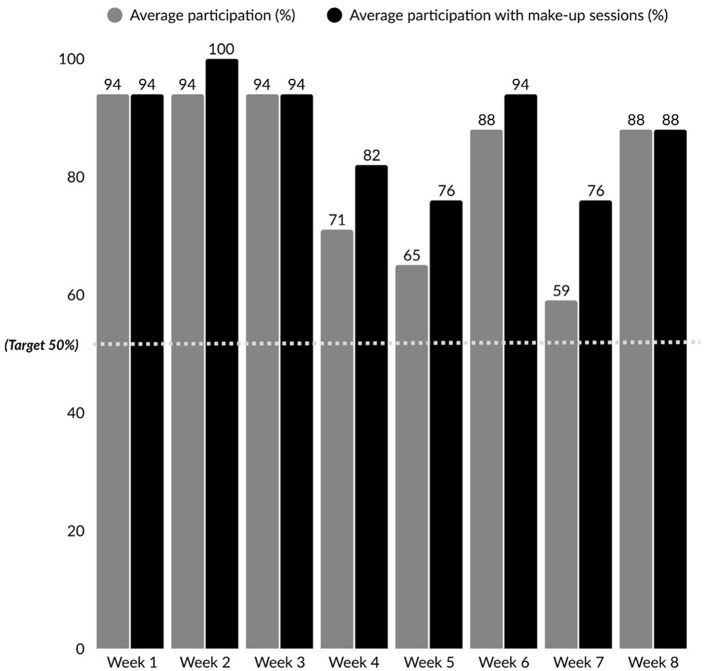
Weekly attendance across intervention sessions.

Of the 18 participants who consented and completed T1, 17 attended at least one session. Among those who initiated the intervention, 10 out of the 17 (58.82%) attended ≥80% of sessions. This was in the GREEN zone. When make-up sessions were included, attendance rates increased to 13 participants attending ≥80% of sessions (76.47%). Figure 5 illustrates average participation from Week 1 to Week 8, including make-up sessions.

### 3.7 Completion rate

Of the 18 participants enrolled, 17 (94.44%) initiated the program. Among those who initiated the intervention, 16 (94.12%) completed all the sessions, resulting in a completion rate of 88.89% among enrolled participants. This met the criterion for the GREEN zone.

### 3.8 Homework completion rate

Overall, participants demonstrated a high rate of homework completion, with average completion rate of 84.82%. As no homework was assigned for Session 1, completion rates from Session 2 through 8 were as follows: 82.35% (Session 2), 82.35% (Session 3), 88.24% (Session 4), 70.59% (Session 5), 82.35% (Session 6), 82.35% (Session 7), and 70.59% (Session 8).

### 3.9 Treatment fidelity

Based on the Facilitator Feedback Checklist, all content outlined in the participant manual was covered in all sessions (i.e., 100% of the sessions), meeting the predefined GREEN zone. With respect to session timing, therapists delivered all participant manual content within the designated 70-min timeframe in 79.17% of sessions. Based on session logs, adherence to the time criterion therefore met the predefined GREEN zone threshold. All 5 sessions lasting beyond the 70 min were in the first group of participants, which also contained the largest number of participants.

### 3.10 . Post-intervention focus group discussion attendance

A total of 16 participants completed the intervention, of whom 15 attended the post-intervention focus group discussion, representing 93.75%. This fell within the GREEN zone.

## 4 Discussion

This study evaluated the feasibility of the *Mental Energy Boost* program targeting CF in PwMS using a *traffic light* system. Most feasibility indicators fell within the GREEN zone, supporting progression to a full trial without major changes. Recruitment duration, was the only indicator that fell within the AMBER zone, indicating a need for correctable adjustments before progressing to a full trial. No feasibility indicators fell within the RED zone. A more detailed discussion of these indicators follows below.

### 4.1 Eligibility

The eligibility rate of 53.84% indicated that more than half of PwMS screened met the inclusion criteria (i.e., presence of objective CF on the PASAT). This rate exceeded the prespecified feasibility threshold and fell within the GREEN zone. This suggests that eligibility criteria were appropriately calibrated to capture an adequate number of PwMS exhibiting CF for this intervention without being overly restrictive. This also indicates that recruitment of PwMS experiencing CF for this intervention is feasible. Notably, the eligibility numbers align with estimated prevalence rates of CF [1 in 2 (~50%)] in PwMS (48, 49), although this may not represent the prevalence of CF in the entire clinic sample, as participation was voluntary and individuals experiencing CF may have been more likely to respond to the study invitation, potentially inflating the observed eligibility rate.

### 4.2 Recruitment uptake

Of the participants who met eligibility criteria, many (85.71%) consented to participate and completed the baseline assessment. This rate surpassed the predefined feasibility benchmark of ≥35% and fell within the GREEN zone. The 35% GREEN threshold was selected *a priori* based on methodological guidance for progression criteria for pragmatic pilot RCTs, where conservative benchmarks are recommended to ensure adequate statistical power for distinguishing acceptable from unacceptable recruitment performance (24). The observed recruitment uptake in the present study was considerably higher than this benchmark, suggesting eligible participants were highly motivated to enroll and engage in the study. High uptake may have also reflected effective recruitment strategies, clarity of study information described in the informed consent, and perceived relevance of the intervention to participants' CF, which they expressed verbally during the screening. The recruitment uptake rate is in line with other behavioral intervention programs for fatigue in PwMS, such as the “Managing Fatigue” study in PwMS which reported a higher recruitment uptake of 96.67% (50). This slight difference may be related to the broader fatigue prevalence and greater recognizability of general fatigue symptoms compared to CF (1, 51).

### 4.3 Duration of the recruitment period

The recruitment period spanned 9 months, placing it within the AMBER zone for recruitment duration. This moderately extended recruitment period can be attributed to several operational and logistical variables. First, of 353 PwMS identified from clinic records, 48 responded to the email expressing interest. It is possible that the clinic records may not have up -to-date contact information, leading to some emails not being delivered to intended recipients. While we anticipated losing a substantial number of people at this stage of recruitment, this was not a formal feasibility outcome we evaluated. However, this should be added as a feasibility outcome in a future RCT. Second, our clinic has several competing studies, which may have limited individuals' availability to participate in this trial. Third, while email recruitment can efficiently reach large numbers of individuals, it carries a high risk of low response rates due to messages not being read. This low screening uptake contributed to the prolonged recruitment period. Future full-scale trials should prioritize face-to-face recruitment during clinic visits, which is likely to yield higher engagement. Fourth, all screening procedures (i.e., reviewing the electronic health records), and all baseline and follow-up assessments were managed by a single research assistant. This restricted the time available for concurrent participant screening while baseline and follow-up assessments were ongoing. In addition, the research assistant was responsible for conducting make-up sessions for participants who missed weekly sessions, further constraining time for recruitment activities. Having additional staffing to share these responsibilities could have reduced the recruitment duration by allowing for more continuous screening and scheduling flexibility.

Recruitment was also affected by clinic closures totaling nearly 1 month during major holiday periods which temporarily halted all research operations. Moreover, recruitment efforts initially targeted only PwMS who had consented to research participation and met preliminary eligibility criteria based on screening via the electronic health record system. Recruitment materials (e.g., recruitment poster) were introduced to potential participants by clinic staff and neurologists only midway through the recruitment period, which limited early exposure of the study to potential participants. Collectively, these factors illustrate how overlapping staff responsibilities, institutional closures, and evolving recruitment procedures may have contributed to an extended recruitment period. In a future full-scale trial, implementing broader recruitment strategies from study outset, and contingency planning for holiday closures would optimize timelines. In addition, in a multi-center trial, more staffing and different clinic closure periods would be anticipated to improve recruitment timelines.

### 4.4 Intervention acceptability

The intervention demonstrated high acceptability among participants who completed the evaluation form, as evidenced by 92.31% of respondents providing a satisfaction rating >5, which fell within the GREEN zone. This level of satisfaction suggests that the program was well received. Similarly, most participants (92.31%) rated the intervention content as helpful, with total helpfulness scores meeting the GREEN zone feasibility threshold. Qualitative feedback corroborated these quantitative findings. Participants reported that they found most strategies (such as physical activity, goal setting, mindfulness, energy conservation, pacing, and mood management) helpful. Many reported adopting resting, energy budgeting, pacing, grounding and breathing exercises, and consistent daily routine into their everyday lives, reflecting successful integration of intervention strategies. Additionally, most respondents reported increased understanding and awareness of CF and improved ability to manage its daily impact, underscoring the program's potential to enhance both knowledge and self-management skills. These findings align with previous research on perceptions of fatigue and fatigue management interventions among PwMS (52), as well as with results from a group-based videoconference-delivered behavioral intervention for fatigue in endometriosis (53).

In contrast, a few participants found certain content, such as context-related and attention-focused strategies less helpful, highlighting the need for tailoring content to individual needs. Recommendations for additional topics (e.g., effective workplace communication regarding CF, assertiveness training, educational resources for family/friends etc.), reflect participants' desire for more comprehensive psychoeducation that extends beyond symptom management to address broader issues affecting their quality of life. Suggestions for program enhancements, including in-person delivery options, follow-up booster sessions, extended implementation period with coaching support, and more interactive facilitation methods, highlight opportunities to optimize engagement, reinforce skill acquisition, and promote long-term adherence. Collectively, these findings support the acceptability and clinical relevance of the intervention while identifying specific areas for refinement to better meet the diverse needs of PwMS experiencing CF.

### 4.5 Adherence

Attendance rates supported the feasibility of the intervention. Nearly all participants (94%) attended at least one session, suggesting good initial uptake and interest in the program. Furthermore, 58.85% of participants attended ≥80% of sessions, exceeding the feasibility benchmark in the GREEN zone. Attendance varied across sessions, ranging from 59% to 94%. In comparison, other fatigue studies have reported slightly lower attendance rates, ranging from 43% to 76% across sessions (53). To mitigate scheduling barriers, participants were offered make-up sessions for any missed content (typically scheduled before the subsequent session) to stay up to date with the program content. When make-up sessions were factored in, the overall attendance rate increased to 76.47%. The inclusion of make-up sessions thus played a critical role in compensating for scheduling constraints, supporting participant continuity, and enhancing overall program attendance.

### 4.6 Completion rate

The high completion rate observed in this study indicates strong feasibility of the intervention. Of the 18 individuals enrolled, 94.44% initiated the program, and 88.9% completed all sessions, surpassing the GREEN threshold. Correspondingly, the attrition rate was 11.11%, indicating minimal dropout throughout the intervention period. This suggests that the program structure, session content, and delivery format were acceptable and accessible for most PwMS experiencing CF. The completion rate observed in the present study exceeds that reported in the “Managing Fatigue” study in PwMS, which reported a 72% completion rate (50). The current intervention may possess distinctive features in the design (e.g., CF-specific content tailored for PASAT-screened participants via targeted strategies) or delivery of its content (e.g., manageable 70-min sessions), which participants found relevant and manageable.

### 4.7 Homework completion rate

Participants demonstrated a consistently high level of engagement with the intervention outside formal sessions, as reflected by an average homework completion rate of 84.82%. Session-specific rates ranged from 70.59% to 88.24%. These findings are comparable to a previous feasibility randomized controlled trial of a behavioral intervention for fatigue in people with IBD, which reported an 80% homework completion rate (46). Although no predefined feasibility criterion using the *traffic light* system was established *a priori* for homework completion, the observed rates suggest that, had such a benchmark been applied, the outcome would have likely fallen well within a GREEN zone. Establishing such a criterion in future studies would provide a helpful benchmark for evaluating participant engagement with homework assignments and could guide intervention refinement, particularly with regard to the practicality of completing homework on a daily basis during the program alongside participants' usual activities and experiences of CF.

### 4.8 Treatment fidelity

High treatment fidelity was demonstrated, with all sessions meeting the predefined GREEN zone threshold for content coverage and the majority of sessions meeting the threshold for session timing. Specifically, therapists consistently delivered all planned session components, achieving 100% adherence to the intervention manual. This finding indicated that the intervention protocol was implemented as designed and that the therapist training, supporting materials (e.g., facilitator manual, checklist, structured session plans) and supervision procedures were effective in maintaining delivery quality across sessions and groups. Similarly, a previous study reported high fidelity, a review of session recordings and fidelity checklists indicating that registered counselors delivered the sessions as intended according to the intervention manual (53). Therefore, the full alignment between the participant manual and session delivery also suggests that the Facilitator Feedback Checklist was a useful and practical tool for monitoring fidelity. This is encouraging for future scalability, as interventions intended for wider dissemination must be deliverable with reasonable precision by different facilitators and across different settings. Ensuring full content delivery also strengthens confidence that any changes observed in participant outcomes can be attributed to the intervention itself rather than to variability in administration. Adherence to the prescribed session duration was also strong, with 79.17% of sessions completed within the target 70-min timeframe, surpassing the study's predefined GREEN zone threshold. Minor deviations from session timing may reflect practical adaptations made to address participant engagement, group dynamics, discussion depth, or logistical issues (e.g., technology, pacing), elements that can naturally vary in behavioral interventions. Overall, the fidelity outcomes suggest that the intervention's core components were conveyed reliably and support the feasibility of delivering the intervention as designed.

### 4.9 Post-intervention focus group discussion attendance

The high attendance rate at the post intervention focus group discussion (93.75%) suggests that most participants were both willing and available to share their experiences following the intervention. In contrast, the fatigue intervention program for individuals with IBD reported a slightly lower attendance rate (70%) for a semi-structured interview conducted face-to-face or over the telephone at the end of their program (46). Notably, their follow-up interview took place 3 months after the intervention, whereas our focus group discussion was conducted within the 2 weeks of intervention completion, which may have contributed to the higher focus group discussion attendance rate. This also suggests that for qualitative data collection, focus group discussion may be a particularly effective option compared with interviews conducted face-to-face or over the telephone, as they can encourage greater participation and engagement.

### 4.10 . Strengths and limitations

Overall, our sample closely reflected a typical Canadian MS population (54), in terms of demographic characteristics. This study was the first to address CF in PwMS through a multidimensional behavioral intervention that accounts for the multifaceted nature of CF. The program was grounded in theoretical models, while also informed by empirical evidence in PwMS. The study protocol was published in advance (6), and feasibility criteria were established *a priori*, minimizing the risk of selective reporting and unsupported progression to a full RCT. An additional strength was that the study attrition rate was low.

However, findings should be interpreted considering several methodological constraints. First, the small sample size and single-center design, with a predominance of individuals with RRMS and a median EDDS score of 2 (range 0–3.5) limits generalizability to progressive MS phenotypes and PwMS with higher levels of disability. Second, although the intervention was developed and adapted based on studies identifying and evaluating factors that impact objectively measured CF (21) and targeted to address objective CF, the intervention was further refined based on a needs assessment that evaluated *perceived* CF (22). This addressed a notable gap in the literature, as no studies to date have systematically examined unmet needs related to CF. Incorporating the lived experiences of PwMS with respect to perceived CF represented a pragmatic approach, as it enabled engagement with a large sample (*n* = 100) prior to intervention to inform feasibility assessment (22). Given that we now have feedback from PwMS through the FGD, the design of a future RCT will be able to address this discrepancy by incorporating feedback from these individuals who have the lived experience of the primary intervention target (i.e., objectively defined CF). Third, while the outcome of the current study focused on objectively measured CF using PASAT, recent evidence from a small sample (*n* = 49 PwMS) suggests that such measures may not always be appropriate due to concerns regarding low test-retest reliability over short intervals (e.g., 5–7 days apart) (55). In contrast, another study has demonstrated adequate test-retest reliability over a comparable interval to that used in the present study (i.e., 2 weeks apart) (Walker et al., submitted)^1^. These inconsistencies may be attributable to methodological differences across studies, which warrant further investigation before proceeding to a future RCT. Fourth, recruitment relied primarily on email outreach to individuals identified through clinic records, which yielded a relatively low response rate (61 of 353 responded, of whom 13 declined, and 9 interested participants were deemed not feasible). This may reflect outdated or inaccurate contact information, as well as the inherent limitations of email-based recruitment (e.g., messages not being read), potentially introducing self-selection bias and limiting the representativeness of the sample. Fifth, the evaluation form was inadvertently not included in the follow-up assessments for the first two groups; once realized, the research team added it for the third group and emailed prior participants for digital completion. Only 8 of 11 participants from the first two groups completed the form, resulting in a total of 13 responses across all three groups. Sixth, a progression criterion was not established *a priori* for the homework completion rate in the study. This was an oversight and should have been considered during the planning phase. Future studies should include a prespecified progression criterion to evaluate the feasibility of the homework, refine its format, or guide the decision on whether to include the homework component in the main trial. Finally, there was heterogeneity in participants' prior behavioral intervention experience. Two participants revealed after study initiation that they had previous exposure to CBT-Insomnia, thus introducing a confound with regard to preliminary efficacy measures (to be reported elsewhere).

### 4.11 . Conclusion

The *Mental Energy Boost* program demonstrated strong feasibility and acceptability as an 8-week group intervention for CF in PwMS, with most indicators meeting predefined GREEN zone thresholds and low attrition. This supports advancement to a full-scale RCT targeting objectively measured CF, with minor adjustments to optimize recruitment duration.

## Author's note

This work was presented as a poster titled “*A Pilot, SingleArm Feasibility Study of a Multidimensional Behavioral Intervention for Cognitive Fatigability in Multiple Sclerosis*” by Tamanna Islam on May 7, 2026, at the International Behavioral Trials Network (IBTN) Conference 2026, in Montreal, Quebec, Canada.

## Data Availability

The raw data supporting the conclusions of this article will be made available by the authors, without undue reservation.

## Footnotes

1 Walker LAS, Murawsky S, Berard JA. Cognitive Fatigability in a multiple sclerosis clinic sample is stable over a two-week test-retest interval: a brief report to inform future interventional studies. *J Neuropsychol*. (2026).
