# Supplementary material for: A pilot, single-arm feasibility study of a multidimensional behavioral intervention for cognitive fatigability in multiple sclerosis: Mental Energy Boost program
Source: Front Neurol. 2026 May 21;17:1823950. doi: 10.3389/fneur.2026.1823950 (PMC13233216; doi:10.3389/fneur.2026.1823950)
Supplement: Supplementary file 1 [file Supplementary_file_1.pdf]

Supplementary Material

Supplementary Table

Appendix A.

The TIDieR-Rehab checklist

Item #	Item	Where located in primary paper	Other† (details)
	**SECTION 1. BRIEF NAME**		
1	Provide the name or a phrase that describes the intervention.	Title, Method and Discussion	
	**SECTION 2. WHY**		
2	Describe any rationale, theory or goal of the elements essential to the intervention.<br>*Essential elements, also known as 'active ingredients', are the core components of the intervention that are expected to be linked to effects or outcomes of interest.*	Introduction and Methods	
	**SECTION 3. WHO**		
3	Describe who the intervention is intended for.	Participants	
	**SECTION 4. WHEN**		
4	Describe when the intervention commenced in relation to the onset or stage of the condition and/or other relevant events.	Participants	
	**SECTION 5. WHAT**		
5A	Materials: Describe any physical or informational materials used in the intervention, including those provided to participants or used in intervention delivery or in training of intervention providers. Provide information on where the materials can be accessed (for example, online appendix, URL).	Intervention Description	
5B	Procedures: Describe each of the procedures, activities and/or processes used in the intervention, including any enabling or support activities.	Procedures	Figure 2
	**SECTION 6. WHO PROVIDED**		
6	For each category of intervention provider (for example, psychologist, nursing assistant), describe their expertise, background and any specific training given.	Procedures	
	**SECTION 7. HOW**		
7	Describe the modes of delivery (such as face to face or by some other mechanism, such as internet or telephone) of the intervention and whether it was provided individually or in a group.	Procedures	
	**SECTION 8. WHERE**		
8	Describe the type(s) of environment(s) where the intervention occurred, including any necessary infrastructure or relevant features.<br>*Environments and their features may include the relevant physical, social, cultural, economic, political and/or systemic context(s) of the intervention.*	Procedures	
	**SECTION 9. HOW MUCH**		
9A	Session(s) duration: Specify the planned session(s) duration of the intervention.	Procedures	Figure 2

9B	Essential elements amount: Specify the planned session(s) duration and/or repetitions of the essential elements of the intervention. *Essential elements amount refers to the time and/or repetitions, within the total duration of a single session, that is spent 'actively' participating in the core components of the intervention.*	Procedures	Figure 2
9C	Frequency: Specify the planned frequency of the intervention.	Procedures	
9D	Intervention length: Specify the planned overall length of the intervention.	Procedures	
**SECTION 10. HOW CHALLENGING**			
10	Describe the approach(es) used to set and monitor the intervention/task challenge level. *Challenge may include the nominal, functional or perceived level of difficulty, effort, physiological intensity or cognitive load of an intervention/task at a given time and may be evaluated using subjective or objective measures.*	Procedures	
**SECTION 11. REGRESSION/PROGRESSION**			
11	Describe the planned regression and/or progression of dosage parameter(s), including when and how. *Dosage parameters refer to the amount (Section 9. How much) and challenge (Section 10. How challenging) of the intervention.*	Procedures	
**SECTION 12. PERSONALISATION**			
12A	Needs: If supplementary strategies were planned to enable the delivery of the essential elements of the intervention in response to specific individual or group needs, then describe what, why, when and how. *Supplementary strategies refer to intervention adjuncts (for example, physical assistance, verbal cueing, props) that must be used by some individuals or groups to facilitate effective participation in the essential elements.*	n/a	
12B	Preferences: If the intervention was planned to be adapted for personal preferences, then describe what, why, when and how.	Procedures	
**SECTION 13. PROTOCOL DEVIATIONS**			
13	If there were deviations in the intervention protocol during the course of the study, describe the changes (what, why, when and how).	Procedures	
**SECTION 14. HOW WELL**			
14A	Plan: If intervention adherence or fidelity was assessed, describe how and by whom, and if any strategies were used to maintain or improve fidelity, describe them.	Treatment Fidelity	
14B	Actual: If intervention adherence or fidelity was assessed, describe the extent to which the intervention was delivered as planned.	Treatment Fidelity	
**SECTION 15. HARMS**			
15A	Plan: Describe the monitoring of adverse consequences. *Adverse consequences include any negative, undesired effects related to the intervention, including physical, mental, social and/or spiritual effects.*	Procedures	
15B	Actual: Describe any adverse consequences, including the number, seriousness and relatedness to the intervention.	n/a	
TIDieR, Template for Intervention Description and Replication.			
